# On the association between rheumatoid arthritis and classical HLA class I and class II alleles predicted from single-nucleotide polymorphism data

**DOI:** 10.1186/1753-6561-3-s7-s33

**Published:** 2009-12-15

**Authors:** Mathieu Lemire

**Affiliations:** 1 Ontario Institute for Cancer Research, 101 College Street, Suite 800, MaRS Centre, South Tower, Toronto, ON M5G0A3 Canada

## Abstract

Using single-nucleotide polymorphisms (SNPs), we sought to predict classical class I and class II human leukocyte antigen (HLA) alleles, and test for their associations with rheumatoid arthritis (RA) in the North American Rheumatoid Arthritis Consortium sample of cases and controls, genotyped on the Illumina HumanHap550 BeadChip. We use publicly available databases of SNP data and HLA data to find SNPs or SNP-haplotypes to be used as surrogates for each HLA allele. To reduce the confounding effects of linkage disequilibrium with the HLA-DRB1 locus, we tested for the association conditional on the presence or absence of a shared epitope allele on the same haplotype as the target HLA allele. Using SNP surrogates, we find that components of the DQ8 serotype (DQA1*0301:DQB1*0302) are associated with RA, irrespective of the presence or absence of a shared epitope allele on their respective haplotypes. Knowledge of the haplotype structure in the HLA region is still necessary for better interpretation of the results.

## Background

The human leukocyte antigen (HLA)-DRB1 locus has long been recognized as a strong genetic risk factor for rheumatoid arthritis (RA), yet it explains less than half of the estimated genetic susceptibility to the disease [1]. Large-scale studies that interrogate the whole genome have uncovered, at strict significance thresholds, genetic risk variants outside the major histocompatibility complex (MHC) [2-4], while also replicating known associations from candidate gene studies [5]. The difficulty in evaluating the role of other candidate loci from the MHC region in the etiology of the disease resides in the strong linkage disequilibrium (LD) and the extended haplotype structure that exists in this highly polymorphic portion of the genome. We seek to evaluate the risk of other HLA loci, by using SNP data and publicly available HLA data, in order to predict and evaluate the effect of classical class I and class II HLA alleles in the North American Rheumatoid Arthritis Consortium (NARAC) case-control dataset [3], as distributed for use at the Genetic Analysis Workshop 16.

## Methods

The NARAC sample consists of 868 cases of RA and 1194 controls, all genotyped on the Illumina HumanHap550 BeadChip, or equivalent. The sample is fully described by Plenge et al. [3]. The sample also includes HLA-DRB1 alleles, typed at various resolutions. The susceptibility alleles at DRB1 tend to share the RAA motif in position 72-74 of the amino acid sequence, an observation that led to the hypothesis of a functional unit, called the shared epitope (SE) [6]. Amino acids found in positions 70-71 provide further refinement of the classification of DRB1 risk alleles [7].

To predict classical HLA alleles from SNP data, we followed the methods described by de Bakker et al. [8]. They typed six class I and class II HLA genes (A, B, C, DQA1, DQB1, and DRB1) in a set of samples that includes the CEU samples from the HapMap (Utah residents with ancestry from northern and western Europe). Most of the HLA alleles they report are at a resolution of four digits. We used this publicly available dataset, combined with SNP genotype data from the HapMap that are in the broad MHC region (chr6: 25, 990, 507...33, 893, 423 [hg18]), and that overlap with the set of SNPs on the Illumina HumanHap550 BeadChip. Using the CEU HapMap data combined with the CEU HLA data from de Bakker et al. [8], we searched for tags for each of the HLA alleles, considering up to three-SNP haplotypes as potential predictors. The best predictor was chosen based on the largest observed *r*^2 ^measure of LD (where, for each target HLA allele, we merged all other alleles at that locus into a single one, to mimic a biallelic locus; the same for multiple SNP haplotypes). To be considered a potential predictor of HLA alleles, a SNP had to be in Hardy-Weinberg equilibrium (*p *> 0.00001) in the set of controls from the NARAC dataset, and had to have a call rate above 95% over all samples. We used the program Tagger [9] as implemented in computer program Haploview [10] to predict the HLA alleles from the HapMap SNP data.

We tested for the association between RA and the class I and class II (non DRB1) HLA alleles, using the SNP predictors as surrogates. Because the DRB1 locus is a strong risk factor for RA, we reduced the confounding effects of LD by performing the analysis conditional on whether each of the two alleles found at the DRB1 locus are members of the SE class of alleles, considering this conditioning argument as if it was a biallelic locus. We used the computer program UNPHASED [11] to perform the conditional tests of association. For each target HLA allele, we report two conditional odds ratios (ORs): these are ORs for the target HLA allele given the presence (SE+) or absence (SE-) of an SE allele on its haplotype. Among the four-digit alleles that are classified as SE+ (according to the classification of du Montcel et al. [7]), those that were actually observed in the NARAC samples only include DRB1*0101, *0102, *0401, *0404, *0405, *0408, and *1001.

The NARAC sample is affected by population substructure, with chi-square statistics reported to be inflated on average by a factor ~ 1.4 [3]. To account for the hidden ancestry of all cases and controls, we computed the spectral decomposition of a covariance matrix between all DNA samples and used its eigenvectors as surrogates for ancestry [12]. The covariance matrix was calculated using a set of ~ 120,000 autosomal SNPs that are at most modestly correlated (pairwise *r*^2 ^< 0.30), a set that excludes SNPs on the short arm of chromosome 6 and on the short arm of chromosome 8 (for reasons explained by Plenge et al. [3]). As in Plenge et al. [3], we found seven outliers by inspecting the eigenvectors associated with the top 10 eigenvalues: their respective entries in at least one eigenvector differed from the mean by more than six standard deviations. We removed these seven outliers from any downstream analyses, and recomputed the eigenvectors. As in Plenge et al. [3], the top three vectors that are statistically significant predictors of case-control status were used as surrogates for the hidden ancestry of all samples, and were used to correct for the effects of population stratification. By using them as covariates in a logistic regression framework, the inflation factor of all association results, excluding results on the short arm of chromosome 6, was calculated to be 1.035. This value is similar to what has been calculated by Plenge et al. [3]. We used these three vectors as potential confounders in UNPHASED.

## Results

We only report the results of the conditional tests of association for those HLA alleles that can be predicted from the set of SNPs described in Methods at an *r*^2 ^> 0.80 (47 out of the 70 non-DRB1 HLA alleles, or 67%), and that moreover show conditional association at the level *p *< 0.001. Table 1 shows, for each HLA allele, its frequency as estimated from the data from de Bakker et al. [8], the SNP or the combination of SNPs that can be used to predict the HLA allele, along with the predictor allele or haplotype, and the strength of the prediction in terms of the *r*^2 ^measure of LD. It also shows the results of the conditional tests of association, including the conditional ORs and their confidence intervals.

**Table 1:** Conditional tests of association between RA and classical HLA alleles through SNP surrogates

					SE-^e^			SE+^f^		
						
HLA allele	Frequency^a^	Surrogate (allele/haplotype)^b^	*r* ^2c^	*p*-value^d^	OR	CI-low	CI-high	OR	CI-low	CI-high
DQA*0301	0.27	rs660895 (G)	0.96	2.13 × 10^-12^	2.11	1.38	3.24	2.11	1.65	2.71
DQB*0501	0.11	rs17533090, rs9275406, rs9275439 (AAG)	1.00	4.30 × 10^-11^	0.33	0.04	2.61	0.43	0.33	0.55
DQA*0101	0.13	rs9268832, rs2395185, rs7774434 (GCG)	0.80	5.12 × 10^-08^	0.58	0.20	1.68	0.48	0.37	0.62
B*0801	0.16	rs3134792 (C)	1.00	1.86 × 10^-4^	1.54	1.03	2.30	3.03	1.27	7.23
DQB*0302	0.19	rs9275312 (G)	0.94	1.96 × 10^-4^	2.23	1.28	3.89	1.38	1.07	1.79
C*0401	0.08	rs9264904 (A)	1.00	1.99 × 10^-4^	0.85	0.56	1.30	0.59	0.44	0.79

^a^Frequency estimated from the data of de Bakker et al. [8]

^b^Alleles or haplotypes used as surrogate for the HLA alleles, with corresponding SNPs

^c^*r*^2 ^measure of linkage disequilibrium between the HLA allele and the surrogate

^d^*p*-value for the two degrees of freedom conditional test of association

^e^Odds ratio and 95% confidence interval for HLA alleles on non-SE allele bearing haplotypes

^f^Odds ratio and 95% confidence interval for HLA alleles on SE allele bearing haplotypes

We find that two class II alleles, DQA1*0301 and DQB1*0302, and one class I allele, B*0801, show significant association with RA irrespective of the presence or absence, on their respective haplotypes, of an SE allele at the DRB1 locus. For DQA1*0301, both conditional ORs are estimated to take the same value, 2.11 (*p *= 2 × 10^-12^). For DQB1*0302, the risk is higher when not combined with an SE allele (2.23 versus 1.38, *p *= 0.0002). The alpha and beta chains DQA1*0301/DQB1*0302 together form the DQ8 serotype [13]. That they are co-associated is thus not surprising. DQ8 has been shown to be associated with RA in humans [14], but this association was thought to be due to LD with DRB1*0401 and *0405 (two SE alleles). Our results are indicative of DQ8 being a risk factor independent of the risk alleles, or non-risk alleles, found at DRB1 (but see Discussion). For the class I allele B*0801, the OR is 1.54 when its haplotype does not contain an SE allele, while it is 3.03 otherwise (*p *= 0.00018). B*0801 is found on the ancestral 8.1 haplotype, which has been shown to carry risk for RA as well as DRB1*03, a non-SE allele [15]. All other HLA alleles from Table 1 show significant decrease in risk only when combined with an SE allele (see Discussion).

## Discussion

Conditioning on the presence or absence of SE alleles on the same haplotype as the test allele at other HLA loci helps reduce the confounding effects of LD with the DRB1 locus, but since different DRB1 alleles, or combinations thereof, show a wide spectrum of risks, this conditioning argument is not sufficient on its own to fully account for DRB1. Knowledge of the haplotype structure in the MHC region is still necessary for a better interpretation of the results. For instance, the apparent protection that seems to be conferred by DQB1*0501 or DQA1*0101 (Table 1) is a mere reflection of the fact that these two alleles are in LD with DR1/DR10 [16], which although they are risk factors for RA, they are not the most prominent ones [14]. Moreover, the classical HLA alleles that we report in the present study are only predicted from the SNP data at hand, sometimes imperfectly, and based on only a small sample (in our case, the HapMap CEU samples). Thus, it is still unclear if the associations seen between DQA1*0301/DQB1*0302 (the DQ8 serotype) and RA truly reflect risks that are independent of DRB1, or rather are artifacts of the measurement errors inherent to any tagging procedure. In terms of the power to detect disease-associated HLA alleles, a penalty is incurred when using SNPs or SNP-haplotypes as surrogates for them, because the sample size required to achieve a given power is inversely proportional to the *r*^2 ^measure of LD between them [17]. Yet, as a proof-of-concept and justification for the more expensive typing of HLA alleles at high resolution, using SNP data and publicly available databases of HLA data to predict classical class I and class II alleles is an efficient method for preliminary evaluation of the role of HLA genes in the etiology of autoimmune, infectious or other relevant diseases.

## List of abbreviations used

HLA: Human leukocyte antigen; LD: Linkage disequilibrium; MHC: Major histocompatibility complex; NARAC: North American Rheumatoid Arthritis Consortium; OR: Odds ratio; RA: Rheumatoid arthritis; SE: Shared epitope; SNP: Single-nucleotide polymorphisms

## Competing interests

The author declares that he has no competing interests.
